# Children’s ability to edit their memories when learning about the environment from credible and noncredible websites

**DOI:** 10.1186/s41235-021-00305-1

**Published:** 2021-05-29

**Authors:** Kim P. Roberts, Katherine R. Wood, Breanne E. Wylie

**Affiliations:** 1 Wilfrid Laurier University, 75 University Ave W, Waterloo, ON N2L 3C5 Canada; 2 Present Address: Carleton University, 1125 Colonel By Dr, Ottawa, ON K1S 5B6 Canada; 3 Present Address: Brock University, 1812 Sir Isaac Brock Way, St. Catharines, ON L2S 3A1 Canada

**Keywords:** Source monitoring, Fake news, Memory development, Technology, Environment, Memory strategy, Internet

## Abstract

One of the many sources of information easily available to children is the internet and the millions of websites providing accurate, and sometimes inaccurate, information. In the current investigation, we examined children’s ability to use credibility information about websites when learning about environmental sustainability. In two studies, children studied two different websites and were tested on what they had learned a week later using a multiple-choice test containing both website items and new distracters. Children were given either no information about the websites or were told that one of the websites (the noncredible website) contained errors and they should not use any information from that website to answer the test. In both studies, children aged 7- to 9-years reported information from the noncredible website even when instructed not to, whereas the 10- to 12-year-olds used the credibility warning to ‘edit out’ information that they had learned from the noncredible website. In Study 2, there was an indication that the older children spontaneously assessed the credibility of the website if credibility markers were made explicit. A plausible explanation is that, although children remembered information from the websites, they needed explicit instruction to *bind the website content with the relevant source* (the individual websites). The results have implications for children’s learning in an open-access, digital age where information comes from many sources, credible and noncredible. Education in credibility evaluation may enable children to be critical consumers of information thereby resisting misinformation provided through public sources.

## Significance statement

Children acquire a large proportion of their knowledge through the internet and social media (Livingstone, 2009). This has been particularly true during the COVID-19 pandemic where many school students are learning exclusively through remote means (e.g., Fiialka, 2020; Garbe et al., 2020; Mabeya, 2020; UNESCO, 2020). Children are actively encouraged by educators to research school projects, and children also rely on the internet for personal matters. Although the internet contains many exceptional websites whose content has been accurately curated, many websites do not contain credible information and/or may have been authored by people who are not experts in a given area. How susceptible are children to accepting such misinformation? Are children able to detect ‘fake news’ and ‘fake content’ on the internet? The question is not trivial—computers are used on all continents of the world in education and the amount of available and accessible information is not likely to reduce in the future. It is essential that the current generation of children are helped to be intelligent consumers of information, and to be able to detect misinformation in websites. In two studies, children aged 7- to 12-years used credible and *not* credible websites to learn about environmental sustainability. There was little evidence of children spontaneously evaluating the credibility of the websites, but 10- to 12-year-olds could ‘edit out’ fake information from their knowledge when explicitly asked to. The results suggest that we cannot let this opportunity to help children resist ‘fake news’ and ‘fake information’ pass by. Researchers should aim to discover more about what processes children need to master in order to be intelligent consumers of digital information.

Children learn from a multitude of sources, for example, teachers, parents, peers, books, television programming, and the internet. Increasingly, school children are encouraged to research a topic by searching the internet for sources of information, that is, websites. In the preschool and early elementary grades when children are aged 4- to 8-years-old, many children (particularly within North America) are shown how to use the internet for research, but their searches are largely guided by the websites chosen by their educators (e.g., Faulk & Evanshen, 2013; the Kindergarten Program, 2016; Long Beach Unified School District, 2013). In contrast, in later grades, when children are aged 9- to 10-years and older, they are expected to take the initiative of finding websites themselves to research a topic.

The sheer amount of information available on the internet is astounding, however, the *quality* of information varies widely. To learn about a medical condition, for example, sources range from highly credible sites like those of the National Institutes of Health or the Mayo Clinic, to websites listing personal experiences as if they were generalized facts, as well as patently false information (e.g., vaccine risks). To maintain the integrity of our knowledge, we must actively evaluate the credibility of each website and the information it contains. Otherwise, we risk having a knowledge base that is inaccurate. In the case of children, without proper constraint, we risk an entire generation that is misinformed or, at the very least, has compromised knowledge. Rather than analyzing children’s internet search strategies, the purpose of the current study was to assess whether children use information about a website’s credibility when they are asked about information from internet sources.

There are two issues that impact the quality of information gleaned from the internet. The first is the accuracy of *source monitoring* (Johnson et al., 1993), which refers to knowing where (i.e., from what source) information originated. The second issue refers to the role of source monitoring during active learning. We discuss each in turn.

### The development of source monitoring

There are many instances when we confuse where we have learned information (see Roberts, 2002, for a review). For example, people can blend or confuse memories of live events and stories (Thierry, 2009), film and narrative about film (e.g., Ackil & Zaragoza, 1995, Thierry & Pipe, 2009), different instances of a similar event (Brubacher et al., 2011; Connolly & Lindsay, 2001; Powell et al., 1999; Zhang et al., 2019), or real-life and suggestions about real-life events (Welch-Ross, 1999). The theoretical role of source monitoring has been used to develop our understanding of eyewitness suggestibility (Gudjonsson et al., 2016), theory of mind development (Bright-Paul et al., 2008), and language devices such as evidentiality in Korean (Papafragou et al., 2007) and Turkish (Lucas et al., 2013) children. Finally, source-monitoring processes have been identified as significant cognitive factors for children with autism (Spitzer et al., 2017), fetal alcohol spectrum disorders (Kully-Martens et al., 2012), and phobias (Klein et al., 2014).

Many factors are responsible for source errors. We are more likely to be confused, for example, between two similar versus dissimilar sources (Lindsay et al., 1991; Roberts & Blades, 1999), when we experience an event repeatedly (Brubacher et al., 2011), and when substantial time has passed and source information is forgotten or cannot be retrieved (Roberts & Powell, 2007). Importantly, source monitoring requires both memorial and metamemorial capacity as it involves reasoning about sources based on the quality of our memories or other knowledge. Non-memorial factors such as executive function (working memory, inhibitory control, set switching, and self regulation) also play an important part. In one study, for example, working memory was related to source monitoring when children aged 4- to 8-years-old were asked to distinguish between a science demonstration and a slide show (Earhart & Roberts, 2014). Even children as young as 2.5- to 3-years-old showed relations between conflict inhibition and source monitoring when children had to decide whether they themselves or a confederate placed animal pieces on a farm (Hala et al., 2016).

Not surprisingly, then, source monitoring is a skill that has a protracted development. Evidence of early source monitoring is concurrent with the accelerated development of the frontal lobes (i.e., the early preschool period; Drummey & Newcombe, 2002). Improvements are seen in both accuracy and breadth in the different types of source decisions until, at least, the early teenage years (ages 10–14; Menon et al., 2005; Raj & Bell, 2010; Ruffman et al., 2001) although most research has been focused on young children aged 10-years and younger (Roberts, 2002).

Understanding the development of source-monitoring skills from basic to adult competencies is informed by Johnson’s Multiple-Entry Modular Memory model (MEM; Johnson et al., 1993; Mitchell & Johnson, 2009). The model outlines two sets of processes: a perceptual set and a reflective set. According to the model, a source is attributed at retrieval as the result of these processes. Memories containing high levels of perceptual detail can lead us to assume that an event was observed rather than imagined (because we assume that such detail would not be present in a memory of an imagined event). Sometimes these decisions are made effortlessly and without awareness. At other times, we need to be more intentionally reflective to attribute a source correctly (e.g., *I couldn’t have seen it because I wasn’t there*). Using these processes enables us to distinguish between ‘internal’ sources (those originating from the self; e.g., thinking, dreaming, self-performed actions) and ‘external’ sources (originating from outside of the self; e.g., observing, hearing).

In the case of learning from websites, however, the source is always external to the self, as websites are presented on a screen (e.g., computer display, smartphone, tablet). Further, although the content and look of websites may differ, the actual medium through which the information is delivered (i.e., the screen) is nearly identical (and not distinctive) when comparing multiple websites. Imagine, for example, a person searching for a topic and then clicking on links to different websites. The websites are listed in the same search, they are both accessed in the same study session, presented on the same device, and so on. Given the similarity of the sources of websites, then, we would expect that a significant amount of development in source monitoring would have to be achieved before it is possible to successfully distinguish websites and information gleaned from them. As children progress through the school grades, there is increasing responsibility placed on them to independently use the internet to find sources of information (children aged 9-years and older).

It has not yet been demonstrated, however, whether children of this age are capable of judging the credibility of websites to maintain integrity of their knowledge. In contrast to much prior research where children choose directly between two sources at the test (e.g., *Was that in the video or the story?* Earhart & Roberts, 2019), using website credibility information involves more indirect use of sources. In other words, children must use source information in the process of choosing and reporting information. Given the difficulty of distinguishing between two different websites, it is important to know whether children can use source information in this way, and subsequently build a reliable and credible knowledge base. Can children later edit out their knowledge, for example, information that they read on a website that contained errors? Can children identify credibility markers spontaneously? Can children use credibility information with adult guidance? And what is the developmental pattern and timing of these processes? We sought to answer these questions in the current set of studies.

### The role of binding processes in source monitoring

It has been demonstrated many times that deficits in source monitoring do not simply reflect a loss of memory (see Raj & Bell, 2010, for a review; Roberts et al., 2016). In studies of repeated-event memory, for example, children can retrieve details from 3 out of 4 different instances of an event (Brubacher et al., 2018). Despite recalling the details, however, children often attribute particular details to the wrong instances (e.g., claiming they did a puzzle during the second instance, when it was actually the third; Brubacher et al., 2018).

Source-monitoring errors in school-aged children are also not caused by a lack of awareness that information is gleaned from different sources. Although preschoolers are still developing the understanding that all knowledge originates from a source (Wimmer et al., 1988), children aged 7-years and above clearly show that they understand the separation of one event from another (Brubacher et al., 2011) and can sometimes identify the differences between them (Brubacher et al., 2011; Danby et al., 2017; Roberts et al., 2015).

An increasingly popular explanation involves the role of binding processes in source monitoring (Bemis & Leichtman, 2019; Burns et al., 2016; Kovacs & Newcombe, 2006; Lloyd et al., 2009; Roberts et al., 2017; Sluzenski et al., 2006). In the work by Newcombe and colleagues, for example, there were few age differences when children identified objects or contextual information (location, scenes) they had seen before, but children aged 3–4 showed impairments relative to older children when recognizing item and context information concurrently (e.g., Did you see the pig in this square?). Newcombe and colleagues argue that these results reflect the difficulty that young children have when *binding* different aspects of experiences together, in this case, the pig and its location. A binding effect was also observed in a study on children’s episodic memory. Specifically, Roberts et al. (2016) found that increased memory for the details of two different events actually corresponded with *increased* source confusion. When the source information was used as a cue to promote reflective attribution, however, source monitoring was improved and the children were less confused (e.g., “Remember that when you wore the cape *it was the time that I was the doctor*”). According to the MEM model, binding content with source is important in source monitoring because the perceptual (e.g., Gestalt processes) and reflective (e.g., noting relations between stimuli) processes act on information to bind content and its context together (Johnson et al., 1993).

Impairments in binding in young children also make sense in the context of other cognitive developments. Between 3 and 6 years of age, substantial progress is made in tasks involving working memory and executive processes (Kanakogi et al., 2012; Zelazo & Muller, 2002). Both the *episodic buffer* (Baddeley, 2000), which binds features from short- and long-term memory, and the MEM framework suppose that executive processes direct attention and resources to particular features—those most characteristic of the to-be-remembered stimuli (Johnson et al., 1993; Ruffman et al., 2001). Some features are processed at the expense of others, so children who are still strengthening their working memory and executive systems may not be able to adequately direct their attention to and encode those features most relevant for later source attribution.

Interestingly, source monitoring, executive functions (e.g., working memory), and binding processes have been localized in similar brain regions, and development of these neural structures coincides with known developmental patterns of cognitive functioning. Much of the neurological evidence regarding the development of source monitoring comes from studies of aging because older adults tend to show lower accuracy when monitoring sources than do younger adults (i.e., paralleling comparisons between young children and adults). For example, Glisky et al. (2001) showed that older adults were less accurate when monitoring sources than younger adults *only* when the seniors had below average frontal function. In investigations of episodic memory with adults, activation of the medial temporal lobe (including the hippocampal regions and the amygdala) is correlated with item and source memory, and the hippocampus is particularly activated when correct source judgments are made (Davachi et al., 2003). Lesion (e.g., Cabeza et al., 2008), ERP (e.g., Wilding & Rugg, 1996), and fMRI studies (e.g., Nolde et al., 1998) provide converging evidence. From fMRI data with older adults in a correct rejection task, brain activations and interactions decreased in the inferior frontal gyrus, supramarginal gyrus, and hippocampus (Tsukiura et al., 2014). Tsukiura and colleagues suggested that the ventral prefrontal region, which is involved in source monitoring, and the inferior parietal region, which is associated with recollection by cooperating with the hippocampus, led to confusion between old and novel stimuli. Parallel neural investigations with children are sparse although the evidence to date reveals a similar neurological profile. For example, Ghetti and colleagues, in their study of middle childhood (aged 6- to 10-years), found that hippocampal and parahippocampal regions of the medial temporal lobe were associated with the retrieval of source-specifying information (Ghetti et al., 2010). Finally, children with autism present with abnormalities of the frontal lobes and related structures and behaviorally show impaired performance on source monitoring and executive function tasks (Spitzer et al., 2017).

### The role of source monitoring and learning

Although source confusions can sometimes be troublesome (e.g., when they affect personal relationships, or a court case hinging on eyewitness testimony), confusing sources may be beneficial in other cases. Specifically, there are demonstrations of an inverse relationship between item and source memory (Ratner & Foley, 2020; Roberts et al., 2016; Sommerville & Hammond, 2007). Ratner and Foley found that when a child and an adult jointly perform a task (making a collage), the children who showed the greatest gains in learning (i.e., item memory) were also the most confused about who placed the pieces on the collage (claiming responsibility for placing pieces on the collage that the adult actually placed; also see Ratner et al., 2002). Naturally, teachers often run their classrooms in terms of one topic at a time using various sources to accomplish learning. As described earlier, children’s memory for the details of two similar events was improved when they were explicitly instructed to do so, but this was at the expense of their source monitoring (remembering what happened, but confusing in which of the events it happened; Roberts et al., 2016). One possibility is that presenting similar sources close together leads children to *blend* the information without reference to source. Indeed, this is one way we can build up knowledge bases in different domains using multiple sources.

The mechanism of *blending* information from different sources can, therefore, be helpful for learning, but there is a caveat when this blending of sources is applied to learning from the internet. While children may trust their teachers to provide accurate information and teachers can carefully choose which sources to use, information that appears in internet searches is not formally vetted and information consumers must actively judge the credibility of websites. Trusting the information on every website would be naïve and likely lead to inaccuracies or omissions in knowledge. Hence, confusing sources may ordinarily help children to build up a knowledge base; but living in the digital world necessitates that children are also able to evaluate the credibility of some sources, such as websites. When an inaccurate website has been identified, for example, a child must identify the origin or source of the compromised information and filter out those details from their knowledge base.

### The current study: learning environmental sustainability information from websites

To investigate whether children can benefit from credibility information about websites, we ran two experiments with 7- to 12-year-olds (an age where most children would be doing at least some independent research using the internet; Holloway et al., 2013). We varied whether children could use credibility information through guidance or spontaneously (without guidance). We also tested whether children can ‘filter’ or ‘edit out’ information that had been encoded, but later discover that the source of this information was not credible. The opposite of ‘credible’ is ‘noncredible’ according to the Merriam-Webster dictionary. For the two website sources, we developed unique (but plausible) websites associated with two different “authors” (confederates; one male, one female) on environmental sustainability. We chose this topic because it is highly relevant to the younger generation who will suffer from the environmental mistakes of previous generations, but also because there is disagreement over some environmental claims. For example, many people display a ‘Christmas tree’ in December but the jury is still out on whether it is better for our environment to buy a natural pine tree or an artificial one. On one hand, natural trees can safely decompose and will re-grow thus sustaining the forest. On the other hand, artificial trees reduce the need to kill a tree each year and can be re-used in subsequent years. Therefore, we could present different ‘facts’ without actually corrupting children’s knowledge of the environment. The important point here is for the reader to have an idea of why environmentalism was a good topic to use to investigate source monitoring of websites.

## Study 1

### Method

#### Design and participants

The design comprised a 2 (Age: 7- to 9-year-olds vs. 10- to 12-year-olds) by 2 (Condition: Credibility information given at test vs. no information given) between-subjects experimental design. In line with research on children’s memories with the same delays as in this study, we set a target sample of 15 per cell (e.g., Buratti et al., 2014; Otgaar et al., 2020; Prabhakar & Hudson, 2019). The final *N* was just shy of this target (*N* = 54). There were 28 7- to 9-year-olds (*M* = 8.08 years, SD = 0.59, range 7.20–9.17, 16 males), and 26 10- to -12-year-olds (*M* = 11.15 years, SD = 0.79, range 10.01–12.87, 11 males). Children were recruited from [University] Family Database and local elementary schools. All institutional and school board ethics committees approved the study, parents provided written informed consent, and children provided verbal assent.

Children first participated in a Search Task using the websites and were tested a week later to see what they remembered (recognition) and the corresponding sources of the remembered details. Credibility condition was randomly assigned at test with the constraint that there were approximately equal numbers of children from each age group in each condition and roughly equal numbers of males and females in each cell. Thus, children who participated in the first session together could be assigned to different credibility conditions at the second session. The independent variables were age and credibility condition, and the dependent variables were recognition accuracy and source accuracy (proportion of details reported from the credible website).

#### Materials

##### The websites

The websites were designed exclusively for this set of studies and contained simple sentences and pictures to teach children about three topics: saving energy, conserving water, and reducing waste. Each had a home page and URL links to other pages as well as videos that could be played. Information on one site had an equivalent message on the other and both sites had the same take home messages. For example, one site claimed that leaving the television on while you sleep can use as much energy as leaving the fridge door open for an hour, while the other site claimed that leaving the television on while you sleep can use as much energy as running a microwave on high for an hour. Both websites claimed “most televisions have a sleep timer which can be set so that the TV shuts off automatically while you’re sleeping, so less energy is wasted—see if your TV has this feature.” The websites were structurally identical and featured 15 target details, 5 for each of the three environmental topics. One website was associated with an adult female and the other with an adult male (two volunteers allowed us to use their image to portray the author of each website). A brief fictional biography of the owners was presented on the home page for each site. Additionally, the websites had either a green background or orange background in order in enhance source saliency. See “Appendix 1” for an example of a webpage within one of the websites. The websites were clearly labeled for the children by referring to them repeatedly as “[Jeff/Linda]’s green site” and “[Jeff/Linda]’s orange site” so that children understood and remembered the labels and the labels could be used to refer to the sources during the interview. See Table 1 for a full list of items and options.

**Table 1:** List of items

Leaving the tv on for 8 h while you sleep can use as much energy as:	Running a microwave on high for an hour	Leaving the oven on for an hour after cooking	Leaving the fridge door open for an hour
Hanging your clothes up to dry can save enough energy to:	Cool your home during the summer	Charge a cell phone 100 times	Heat your home for the winter
An energy saving dishwasher can save as much energy as would be created by:	1000 skips with a skipping rope	1000 jumping jacks	1000 hoolas with a hoola-hoop
Closing the damper on a fire place can save up to:	$500 per decade	$500 per month	$500 per year
A computer monitor should be turned off if it's not going to be used for:	10 min or longer	Overnight	Half an hour
Per flush, a low flow toilet can save enough water to fill a:	Medium cooking pot	Small cooking pot	Large cooking pot
A regular flow toilet can be made into a low flow toilet by putting _____ in the toilet tank:	Large pop bottle	Glass jar	Salad dressing bottle
Leaving the tap running while you brush your teeth can waste enough water to fill a:	20 child-sized rain boots	20 medium-sized winter boots	20 adult-sized running shoes
A leaky faucet could fill an average sink in just:	1 lunch break	1 night of sleep	1 day at school
A 5 min shower with a standard shower head uses ____ of water:	A whole bathtub (150 L)	A whole hot tub (1000 L)	A children's swimming pool (500 L)
Recycled pop cans can be made into:	A wheel chair frame	Aluminum water bottles	A bike rack
Recycled glass jars can be made into:	Plates and bowls	Mirrors	Windows
Recycled plastic bottles can be made into:	Toys	Backpacks	Water wings
1 tree can remove up to ___ of CO_2_ from the atmosphere over the life of that tree	100 airplanes full	100 school buses full	100 hot air balloons full
Composting can reduce household garbage up to:	80%	60%	50%

The presentation of the websites was counterbalanced so that the site seen in green first by half of the children was seen in orange first by the other half of the children. In addition, the order of website author was counterbalanced so that half of the children saw “Jeff’s site” first and half saw “Linda’s site” first. Finally, the website that was described as noncredible for some of the children was equally often Linda’s or Jeff’s website. Thus, each website was seen equally often as green or orange, each colored site was seen equally often first or second, and each website was equally as often described as the noncredible website.

##### The test

The interview consisted of a multiple-choice quiz asking about details from the websites. There were 15 questions and each answer slate comprised three options: one from each of the two websites and one from neither website (i.e., a distractor). For example, children were asked what recycled pop cans could be made into; the option from the one website was a wheel chair frame, the option from the other website was a bicycle rack, and the option from neither site was aluminum water bottles (see “Appendix 2” for the full test). Distractor items were designed to ensure that children would not be able to utilize general reasoning and prior knowledge to correctly reject new items. For example, children were tested on specific information (e.g., leaving the tap running while you brush your teeth can waste enough water to fill 20 child-sized rain boots), rather than general tenets of environmental sustainability (e.g., it’s important to turn your tap off when brushing your teeth; see “Appendix 3”). Further, the placement of the distractor items (response option a, b, or c) was randomly distributed throughout the test to avoid test format as a factor in response selection.

#### Procedure

##### Session 1: the study and search task

A Research Assistant (RA) asked children if they would like to do some activities and learn about the environment using a laptop. Following children’s assent to participate, they were taken in groups of up to 3 to another room where they participated, using separate computers, in the website search task. Trained RAs opened the websites prior to participants’ arrival and followed a script to introduce the activities (“Hi, my name is [RA name] and today we are going to explore two websites to learn about the environment. This is [Jeff’s/Linda’s green/orange] website and this is [Jeff’s/Linda’s green/orange] website.”). The session was referred to as the ‘Environmental Website Activities with [RA name])’ to facilitate remembering the event session at test. All children viewed both websites during the event session. The purpose of the search task was used to ensure that all children attended to and integrated the information from the websites.

The search task took approximately 30 min and involved individually answering three specific questions from each site. For example, children were directed to one of the websites to see how much energy could be saved by hanging their clothes up to dry. They then searched the specified site for that information. The RAs helped the children as needed, but encouraged them to find the answers on their own. For example, if a participant demonstrated difficulty in finding the information within the websites, the RA might say, “Hmm…this question seems to be about water [e.g., leaky faucet], let’s see if we can find an answer on the Water page” (directing the participant to the appropriate page of the website). In educational contexts, information from different sources is combined together; thus, the children next completed a ‘knowledge integration’ task where the children had to use both sites to answer six more questions (see “Appendix 2” for full test). These six questions were of the form: “Name three things that …” and were to be filled in with one answer from each site and one answer that they came up with using information already in their knowledge bases. RAs ensured that all child-generated responses (e.g., plastic bottles can be made into pants) were unique to the child and not from either of the websites. Any assistance provided during this task was done in such a way that the person assisting did not become an additional source (i.e., specific examples were not provided). This was done to increase blending of information from the target websites in an ecologically valid way. This procedure also made it possible to verify that the children paid attention to and encoded the target details.

##### Session 2: the interview

The RA introduced the task by saying, “It is my job to find out what children remember, so I’d like to ask you some questions about what happened the day you did the Environmental Website Activities with [RA name]).” The RA asked whether or not each child remembered the website activities and all children did; therefore, no child was excluded. Half of the children were told that one of the people associated with the websites (either Jeff or Linda) made some mistakes on his/her website and the children were asked to disregard information from that site (see “Appendix 3”), and only choose options from the other person’s website when answering the test questions. The remaining children were only told to choose the most correct answer and were not given any other information, thus, providing a measure of how often individual details are chosen with no regard to which website it came from. After giving the instructions, the RA provided participants with a hard copy of the 15 multiple-choice questions. The RA instructed participants to circle the letter (a, b, or c) that corresponded to the answer that they thought was the most correct and instructed them to complete the task independently. If participants requested assistance during the test phase, the RA reminded them to select whichever answer they thought was the most correct.

##### Scoring

Given the simplicity of the task, children’s responses were recorded directly on the test forms during the interview session. The forms were later double coded and there was 100% agreement between raters. Responses could be coded on the basis of the detail origin so that a detail that came from: (1) the website deemed credible was referred to as a *credible website detail;* and (2) from the website deemed noncredible (an *noncredible website detail*); and (3) a *false alarm* if the detail was not present in either of the websites. Note that children in the control group were not given information about credibility and so technically reporting details from either website is correct for them. The control group responses provide the probability of choosing a detail from a website in the absence of credibility information. Proportional scores of each of these categories were calculated to allow for a small number of missing responses. Responses from children who were not provided with credibility information were coded using the same credible website as those in the credibility condition. This allows comparison between choices from the credible website with and without instruction.

### Results: study 1

#### Preliminary analyses

Even though we had thoroughly counterbalanced the website stimuli, we explored whether there was any bias to choose a particular website. Analyses of the key variables were run on (a) the author of the credible website (Linda or Jeff), (b) the author of the website that was presented first, and (c) which website was considered noncredible. There were no differences, *ps* = *ns*. Analyses were also run on participant gender and there was no difference in scores between boys and girls, *ps* = *ns*. *Fs* for all tests ranged from 0.02 to 1.9.

#### Main analyses

##### Overall recognition differences

Before analyzing source monitoring between the web sites, it was first necessary to verify that children remembered the information from the websites. Without considering source, recognition of information from the website and correct rejection of distracters was impressively high. Proportion scores for the number of correct recognitions (out of the total number of questions answered) were calculated for each participant. A between-subjects analyses of variance (ANOVA) was run using Age group (7- to 9-year-olds, 10- to 12-year-olds) and Credibility Condition (given at test, or not) on the *hits* for the details in each website (regardless of whether the responses were correctly attributed to source later). There was a main effect of age, *F*(1,96) = 13.20, *p* < 0.001, *η*^2^*p* = 0.124, because older children recognized more information than did younger children (*M*s = 0.98, 0.94, and SDs = 0.05, 0.08, for the older and younger children, respectively). There was also an Age x Condition interaction, *F*(1,96) = 4.97, *p* = 0.028, *η*^2^*p* = 0.05, because the age difference (older children recognizing more information) was present only when no credibility information was provided, *t*(41) = − 0.412, *p* < 0.001, *Cohen’s* *d* = 1.295 (large effect), CI 95% [− 0.11, − 0.04]. The age difference was not evident when credibility information was provided, *t*(52) = − 1.02, *p* > 0.30. See the ‘Recognition Correct’ column in Table 2 for means and standard deviations.

**Table 2:** Mean (standard deviation) recognition and source accuracy scores from Study 1 by age and credibility instruction condition

Condition	Recognition correct	Credible website	Noncredible website
7- to 9-year-olds			
Credibility (at test)	.95 (.07)	.53 (.14)	.43 (.12)
No credibility	.92 (.08)	.48 (.12)	.44 (.12)
Total	.94 (.07)	.50 (.13)	.43 (.12)
10- to 12-year-olds			
Credibility (at test)	.97 (.06)	.58 (.188)	.40 (.18)
No credibility	.99 (.02)	.50 (.12)	.50 (.13)
Total	.96 (.07)	.54 (.17)	.44 (.17)

##### Source accuracy

Proportion scores for the number of details from the credible website (according to the website deemed to be credible and out of the total number of questions answered correctly at test) were calculated for each participant. A 2 Age × 2 Credibility condition ANOVA was run on the details from the credible website. There was no age difference, *F*(1,96) = 1.33, *p* = *ns*, but there was a main effect of Condition, *F*(1,96) = 4.92, *p* = 0.03, *η*^2^*p* = 0.05. Children given credibility information at test reported proportionally more details from the credible website (*M* = 0.55, SD = 0.18) than did children who did not receive any credibility information (*M* = 0.49, SD = 0.12)*.* The Age x Condition interaction was not significant, *F* < 0.20, *ns.* The means and standard deviations are in Table 2 (Credible website column).

##### Chance comparisons

Next, each age group was separately analyzed to see whether the proportion of deails they reported from the credible website differed from chance. Although chance is technically 0.33 because there were three options on the test (not seen, seen on website A, seen on website B), chance was set more conservatively at 0.50 given that recognition scores were so high (i.e., very few recognition misses). Thus, the comparison chance score reflects that, once a detail was recognized, children chose between *two* websites. The chance scores of children in the control condition should be no different from chance given that they had no instruction as to which website to draw details from. Regarding children in the control condition, as expected, the scores of the 7- to 9-year-olds did not differ from chance, *t*(25) = − 0.963, *p* = 0.346, and neither did the scores from the 10- to 12-year-olds, *t*(19) = − 0.109, *p* = 0.915. Only the scores from the 10- to 12-year-olds in the credibility condition were above chance, *t*(25) = 2.08, *p* = 0.006, *Cohen’s d* = 0.64, (medium effect), CI 95% [0.0009, 0.15] (7- to 9-year-olds: *t*(27) = 1.123, *p* = 0.271) 0.7- to 9-year-olds in the credibility or control condition, *t*(27) = 1.12, *p* > 0.20, but the 10- to 12-year-olds scored above chance.

### Discussion: study 1

Source misattribution has been treated as problematic in most previous studies of source monitoring. There is one sense, however, where forgetting or confusing sources can be considered adaptive, such as in educational settings where the main goal is to build up a knowledge base. Highlighting the similarity between different teaching materials can boost memory of the information, yet similarity is detrimental with respect to source accuracy. In these situations, it may be an advantage to lose source information and synthesize the retained information together to build a coherent depth of understanding. In studies by Ratner and colleagues, for example, children who inaccurately claimed that they placed pieces on a collage that a confederate had actually placed, showed improved recall and organizational skills even though the sources—child versus adult—were confused (Ratner & Foley, 2020; Ratner et al., 2020). In a more recent study, and in line with Foley and Ratner’s theoretical stance, improving children’s encoding of information *increased* source confusions, presumably because they remembered more information per se while largely ignoring the sources (Roberts et al., 2016). Indeed, in the present study, children needed to search for information from both websites to accurately complete the task (thus they were encouraged to pay attention to the information rather than the sources). The impressive recognition performance also supports the idea that children encoded the information well.

Credibility information clearly made a difference to the accuracy of children’s source monitoring. Children who had been told at the test that one of the websites was credible, and the other not, were able to use this information during the test. This was evidenced by children accurately reporting information from the credible website more often than the children who were given no information about credibility. Additionally, inaccurately reporting information from the noncredible website (source errors) and mis-recognizing distracters was decreased when credibility information was given versus not given at all. These results support the notion that children *can* use qualitative information about the reliability of websites to inhibit reporting noncredible information even though they clearly remembered the noncredible information. Indeed, even very young children can distinguish between reliable and unreliable sources of evidence. For example, preschoolers aged 5- to 6-years-old will trust an informant who previously reported accurate information over an informant who previously erred (e.g., Jaswal et al., 2010; Mills et al., 2011). However, in the current study, children had no prior experience with these websites and had actually learned and remembered the information from both websites. This type of credibility judgment is arguably more difficult than when there is a history of experiences to rely on. Indeed, the children in this study were given the credibility information just moments before the test (and therefore a while—a week—after learning the information). This research highlights novel ways that sources must be evaluated, ways that extend well beyond the preschool and elementary period.

The developmental pattern in the results was affected by children’s exposure to credibility information about the websites. Even though we can be confident that children had encoded information from the two websites (as seen by the impressive recognition and rejection scores), there were clear age differences in recognition accuracy. Age differences, however, largely disappeared when children were instructed to use information from only one website, the credible one. Specifically, the older children were better at recognizing information from the websites and correctly rejecting distracters (information not presented in either website) than the 7- to 9-year-olds were, but only when no credibility information was presented. When credibility information was given, the 7- to 9-year-olds were as accurate as the older children. The effect of credibility information on age differences in source accuracy was less clear. On one hand, there were no age differences in the number of times correct sources were reported, but only the 10- to 12-year-olds who were given credibility instructions were able to select the credible website above chance levels. This suggests that all children found the task difficult, but that there is substantial progress in source evaluation between the ages of 7- to 12-years. Previously researchers have ignored source-monitoring development past the ages of 8- to 10-years old, believing that such development was complete. It is clear from these results, however, that the way sources are used to build up a knowledge base may not be complete until the tween or even teen years. These results make sense from a developmental neurology perspective. As the frontal lobe develops, reasoning skills and inhibitory control become more reliable, both of which can aid in source monitoring (Nolde et al., 1998).

It appears that children can utilize credibility information to report credible information, however, the extent of their ability to use this information appears to be limited. Although children who were given credibility information at test reported more details from the credible website and fewer from the noncredible website compared to children who were not given any credibility information, when compared to two controls (the no-credibility condition, and chance), most children barely reached 50% accuracy with source attribution. The fact that these children clearly remembered the information but had difficulty attributing pieces of information to the respective websites raises some concern. If unreliable information is learned, it seems that children (at least those of the ages in this study) cannot later spontaneously remove or ignore the noncredible information very well. As there is a wealth of websites that are not officially sanctioned and checked for accuracy, this poses a great risk for the quality of information children might be learning from the internet.

Nevertheless, it is still important to investigate whether children can use their limited source-monitoring skills to preserve an accurate knowledge base. In a world where the quality of information varies dramatically from website to website, it is essential that we are able to judge the quality of what we see and read. Although, as in the simulation in Study 1, we sometimes do not discover the credibility of information until later (e.g., a professor grades a completed essay and comments that the student should not use Wikipedia sites that are not validated), it is more practical to be able to use credibility information as one is browsing internet sites. Time wasted on websites that provide information that cannot be used could be better spent reading from and viewing credible websites. This is the focus of Study 2.

Finally, it is possible that the effect we found in Study 1 was a Type I error if the study was underpowered (recall that the target sample was not reached). Thus, Study 2 also provides an opportunity to see whether the effect found in Study 1 could be replicated.

## Study 2

Similar to Study 1, in Study 2, children studied the two different websites and were tested a week later to see what they remembered. In contrast to Study 1, during the learning phase, one of the two websites contained ‘clues’ to its untrustworthiness in the form of spelling mistakes, inconsistencies, contradictions, and so on (see “Materials” section). The credibility instructions mimicked those in Study 1, that is, half of the children were warned of these mistakes (i.e., provided credibility information) at the test; the remaining children were not given any credibility instructions at all. In other words, all children viewed two websites, one with credibility ‘clues’ and one without; 1 week later, half of the children were told about the untrustworthy website at test. If there is no improvement in source accuracy for those children who are not explicitly provided credibility information at test (given the same instructions are control group in Study 1), it suggests that children (of the ages in this study) can spontaneously monitor credible aspects of websites and later utilize this information to more often report credible information.

### Method

#### Design and participants

A new set of children was recruited for Study 2 and comprised 71 children aged 7- to 9-years (*M* = 7.92, range = 7.1–9.19, *n* = 29) and 10- to 12- years (*M* = 11.23, range = 10.1–11.99, *n* = 42). Initially, 78 children participated but 4 were excluded from the study because they were absent at the second session, and 3 did not complete the task. A post hoc power analysis with the program G * Power (Erdfelder et al., 1996) determined that with *α* set at 0.05, there was an 80% chance of detecting a medium-large effect. Participants were randomly assigned to conditions; with the same constraints as those in Study 1. Participants were recruited from local schools in the same school district as in Study 1. Permission from the relevant institutional Ethics Board and School Board was obtained. Written informed consent from parents was given prior to the study, and verbal assent by children was given prior to both sessions (once before the event session and once before the interview session).

#### Materials and procedure

The search task, test, delays, and counterbalancing measures were identical to those used in Study 1. Credibility information was provided at the test for half of the children, whereas the remaining children received no credibility warning. The same websites as in Study 1 were used except that the website that was deemed noncredible was modified slightly so that it contained errors which served as a proxy for credibility. Credibility cues included spelling mistakes (e.g., skool, folow, recyckled), grammatical (e.g., planting 1 trees, 10 bags of garbages to 2 bages of garbages) and punctuation errors, poor word choice (e.g., composting adds soil nutrients and minuses waste), missing words (e.g., Linda that a leaky faucet…), conflicting information, broken external links, lack of external sources, and decreased level of authority in the author (“teaches science” vs. “in sales”). This was done to provide “clues” or indicators that could be used to distinguish a credible source from a non-credible source at test.

### Results: study 2

#### Correct recognition

To verify that children remembered the website, the proportion scores for the number of correct recognitions (out of the total number of questions answered) were calculated for each participant. Recognition was impressively high as it was in Study 1. A between-subjects ANOVA was run using Age group (7- to 9-year-olds, 10- to 12-year-olds) and Credibility Condition (given at test, or not) on the *hits* for the details in each website (regardless of whether the responses were correctly attributed to source later). There was a main effect of age, *F*(1,59) = 6.40, *p* = 0.014, *η*^2^*p* = 0.098, because older children recognized more information than did younger children (*M*s = 0.93, 0.86, and SD*s* = 0.08, 0.11, for the older and younger children, respectively). There were no other effects. See the ‘Recognition Correct’ column in Table 3 for the full set of means and standard deviations.

**Table 3:** Mean (standard deviation) recognition and source accuracy scores from Study 2 by age and credibility instruction condition

Condition	Recognition correct	Credible website	Noncredible website
7- to 9-year-olds			
Credibility (at test)	.86 (.13)	.53 (.16)	.32 (.09)
No credibility	.87 (.08)	.48 (.11)	.36 (.15)
Total	.87 (.011)	.51 (.14)	.34 (.12)
10- to 12-year-olds			
Credibility (at test)	.93 (.07)	.57 (.14)	.36 (.128)
No credibility	.92 (.09)	.46 (.16)	.45 (.15)
Total	.93 (.08)	.52 (.16)	.41 (.14)

#### Source monitoring

To assess source accuracy, we conducted a 2 Age × 2 Condition (no credibility information at test, credibility information at test) ANOVA on the total number of details reported from the credible website). There was a marginally significant main effect of condition, *F*(1, 59) = 3.88, *p* = 0.05, *η*^2^*p* = 0.06, because children who were given credibility information at test (*M* = 0.56, SD = 0.14) reported more details from the credible website than those not provided with information (*M* = 0.47, SD = 0.15). The interaction was not significant, *F*(1, 59) = 0.443, *p* = 0.508, and there was no main effect of age, *F*(1, 59) = 0.045, *p* = 0.832. See Table 3 for the full set of means and standard deviations.

Chance analyses were conducted on the proportions. The scores from each Age group × Credibility condition were compared to the conservative 0.50 (see “Results: study 1” section for an explanation). Responses from the 7- to 9-year-olds in either credibility condition did not differ to chance, *p* = 0.64. However, the scores of 10- to 12-year-olds who had received a warning were significantly higher than chance, *t*(20) = 2.27, *p* = 0.034, CI 95% [0.01, 0.13].

#### Could children spontaneously identify the credibility markers?

The design of Study 1 and 2 differed only in whether both websites were equally as credible (Study 1) or only one website was credible (Study 2). In both studies, credibility information was given at the test or not at all. To decipher whether the children in Study 2 were spontaneously able to identify and use the credibility clues *without* being given any explicit credibility information, we compared the number of details from the credible website recalled by the children in the no credibility conditions of Study 1 and Study 2. If the children who studied the low-credibility website were more accurate than those who studied two equally credible websites, it suggests that children are able to spontaneously identify features of websites that are clues to its low credibility.

The proportion of details from the credible website were entered into a 2 (Age) × 2 (Credibility Study: both sites credible [Study 1], one site credible [Study 2]) ANOVA. There was a main effect of study, *F*(1,72) = 5.68, *p* = 0.02, *η*^2^*p* = 0.07, which was qualified by an Age × Study condition interaction, *F*(1,72) = 8.99, *p* < 0.01, *η*^2^*p* = 0.11. Independent-samples *t* tests were carried out to compare credibility condition separately for each age group. The 7- to 9-year-olds reported more details from the credible website when the two websites were both credible than when only one website was credible (*M*s = 0.54, 0.42, and SDs = 0.04, 0.13, respectively), *t*(33) = 4.31, *p* < 0.001, *Cohen’s d* = 0.51, CI 95% [0.07 0.18]. Credibility condition had no effect on the reporting of credible details by the 10- to 12-year-olds however, *t*(39) = − 0.42, *ns* (*M*s = 0.50, 0.52, and SDs = 0.01, 0.15, respectively). The full set of means is presented in Table 4.

**Table 4:** Mean (standard deviation) source accuracy scores from Studies 1 and 2 by age and website credibility condition

Age	Condition	Credible website details
7- to 9-year-olds		
	Study 1: two equally credible websites	.54 (.04)
	Study 2: one credible; one noncredible website	.42 (.13)
	Total	.49 (.10)
10- to 12-year-olds		
	Study 1: two equally credible websites	.50 (.01)
	Study 2: one credible; one noncredible website	.52 (.15)
	Total	.51 (.11)

No credibility instructions were given to the children whose means are presented. Study 1 comprised two equally credible websites; Study 2 comprised one credible website and one website with clues to its lack of credibility

Finally, analyses were run to directly compare the two credibility conditions to chance. Only the 10- to 12-year-olds in the two-credible-website condition (Study 1) answered more accurately than would be expected by chance, *t*(20) = 2.27, *p* = 0.034, *Cohen’s d* = 0.51, CI 95% [0.01, 0.13].

### Discussion: study 2

The effects of credibility information on source monitoring found in Study 1 were replicated in Study 2. Children’s source monitoring was improved when warned just before the test not to consider any information from the website that was not credible (compared to when no credibility information was presented). This effect was evident when comparing source errors with correct source attributions. There was no difference in correct attributions and errors pertaining to the websites by children who were not warned while those who were warned reported more details from the credible versus the noncredible website. Once again, the older children in particular seemed to benefit from credibility instructions more than the younger children. Only children in the 10–12-year-old group who were warned about website credibility scored better than chance. We also wondered whether children could independently deduce that a website was not credible based on mistakes within it. In this analysis, the 7- to 9-year-olds reported more details from the credible website when both websites were visually as credible as each other versus when one website contained visual errors. Even then, however the 7- to 9-year-olds did not report credible details above chance. When these analyses are considered together, it suggests that 7- to 9-year-old age range is a sensitive and dynamic time for using source credibility. Some analyses imply sensitivity to source clues (7- to 9-year-olds were more accurate when both websites were equally credible compared to when one of the websites had clues to its lower credibility); yet, the warning per se did not improve their accuracy and their scores were no different to chance. Given the novelty of this research, more sensitive measures of source credibility may be created in future research to provide a fuller picture of what processes are changing in this age range.

Given that the participants were matched by age, gender, neighborhood, socio-economic status, and the procedure of the studies was identical each time, it can be assumed that the credibility manipulation (one site containing cues to credibility) did not improve accuracy. Its effect, however, was negative on the test scores of the 7- to 9-year-olds. While an unusual result at first glance, these results are similar to those reported by Roberts et al. (2016) who found that paying attention to differences between sources can sometimes result in lower accuracy when monitoring the sources. In both the Roberts et al. (2016) study and in Study 2 here, the source task can be considered difficult because the sources were overall quite similar and the accuracy scores reflect task difficulty. Hence, a plausible explanation might be that the differences between the two websites in this study distracted the younger children from *binding* the source with the information. Thus, children remembered the information well, but at the expense of source monitoring. This process is optimal when learning information is key (as in Ratner and Foley’s work), but falls short when there is a high need to monitor the trustworthiness of those sources.

## General discussion

Children learn a massive amount of information from the internet, both formally (e.g., in education settings) and informally (personal interests), most of which is unfiltered for content. The internet is the place children go to find out information although most of the content is unfiltered. Unfortunately, not all websites contain credible information. Noncredible websites can spread misinformation such as ‘fake news’. If children are not aware of the credibility rating of websites, there is a very real danger that fake information will be incorporated into their knowledge base. This is particularly concerning at the time of the COVID-19 pandemic because literally millions of children are learning solely through remote access and not in person with their teachers.

The findings of the studies presented here clearly show that children aged 7- to 12-years old seldom use information about credibility when learning from websites. The 10- to 12-year-olds, however, were receptive to explicit information about website credibility and were able to apply that knowledge. Specifically, these older children reported less information learned from the noncredible website than the credible website if given credibility information. Importantly, this effect was replicated in both experiments regardless of when the information was presented. This suggests that even if information from noncredible websites is encoded, 10- to 12-year-olds can “edit out” the inaccurate information if told at test to ignore information from a particular website.

A plausible explanation for the older children’s use of credibility information is that these children were able to link information with its source (i.e., which website) and apply that information as they are building their knowledge base. The ‘linking’ process is typical of *binding* mechanisms where information and its source are bound together during the encoding process. As the 10- to 12-year-olds were able to correctly assign information to its source at the test phase (1 week after encoding), it suggests that information and source continued to be bound together over the delay. If information was not bound to the credible website, children would score at chance and make almost as many source errors as accurate source attributions, like the explicitly warned 7- to 9-year-olds did.

Unlike the older children, the 7- to 9-year-olds did not benefit from explicit credibility warnings. This may not be a domain-free problem with binding, because in other circumstances, same-aged children bind sources more effectively than younger children. It has yet to be elucidated what impact tasks, test material, and delays differentially impact binding. What it does make clear, however, is that source-monitoring development is not complete by age 9 years as previously thought. These children were not able to reject ‘fake’ information even when told to. The 7- to 9-year-olds may not have bound source and its corresponding information together, or perhaps they could not effectively regulate the reporting of noncredible information. Another possibility is that they did bind source and its information at encoding but, after a delay, connections between information and its source have deteriorated such that it is no longer possible to edit out the noncredible information from the noncredible website.

These results provide tentative evidence about why adults are susceptible to believing fake news. Given the developmental pattern seen in the current studies, adults should be able to use credibility information (at least explicit information) to shape their knowledge. Even if adults are able to bind source and information, and later use this to edit their knowledge base, it is possible that decisions about what sources are credible vary between individuals. If information is presented from a source that is perceived to be credible (e.g., a president of a country, a particular news station or media outlet, Wikipedia), the corresponding information may be retained. Other people, however, may consider the exact same sources to be noncredible and are able to reject the corresponding information or at least use information about source credibility some time later.

When no credibility information was provided, and when children did not spontaneously pick up on cues to credibility, the credible and noncredible websites were confused. This may generalize to other domains where credibility information may mitigate the use of fake information, for example, when determining whether a piece of information came from a witness or a rumor, or whether a ‘fact’ came from an authority versus an advertisement.

Further research studies with larger sample sizes than those provided here will give a better sense of how warnings provided to children of different ages can promote the editing of false or noncredible information in memory. A future study could also consider how children’s search strategies are affected by source credibility. It will also be profitable in future research to consider how the timing of the warning might affect source-monitoring processes differently. In studies of eyewitness misinformation (Melnyk & Bruck, 2004; Roberts & Powell, 2007; Roberts et al., 1999), children are more susceptible to accepting misinformation about an event if the false details are presented a while after the event when memory for the event has decayed, and less susceptible when presented immediately after (possibly because they are able to do a fast recognition analysis of whether the detail is familiar or not).

The surge of information available to us and our children has forced us to be “critical consumers of information”. Just as people may ‘shop around’ for the best deals, or goods made by a reputable (credible) company, people need to be critical about where they get their information from. It is unlikely that the internet will stop providing easily available and accessible information, and that also means an increase in noncredible websites. We are in the fortunate position, however, that we can bind information and its source and use it to actively monitor our knowledge about the world. A worthy goal, now, is to figure out how to enable children to be aware of the sliding scale of credibility so that they are not victim to fake news and the like.

## Conclusions

The current generation of children has a major advantage to previous ones in that information is readily available at the click of a button. An internet search can produce dozens of websites on a particular topic. Some websites contain accurate information, and others have false information, as in the case of ‘fake news’. The results presented here, however, show that elementary-aged children do not filter information from websites according to its credibility. Unless explicitly told that the information from a website was not credible, 10- to 12-year-olds blended information from websites with accurate details with those deemed to contain fake information. Results with younger children were even more concerning. Seven- to 9-year-olds used information from both credible and noncredible websites to answer a test even if they were informed not to use anything that they had read on an noncredible website. Given the increased rate of accessible and available information on the internet, and the existence of misinformation and ‘fake news’ displayed on some websites, there is a very real societal concern that children will base their current and future decisions, opinions, likes and dislikes on information that is inaccurate at best, but often deliberately false. The challenge we now face is how best to enable children to filter information and consider the credibility of the source when exposed to vast amounts of information on the internet.

## Data Availability

The datasets generated and/or analysed during the current study are not publicly available because permission was not requested during the informed consent procedure. The departmental and institutional REBs are currently debating procedure on this issue. However, as the data are anonymous, the corresponding author on reasonable request will seek permission from the REB to share the de-identified data files.

## Appendix 2: Questions at the study session

Use _________ website to fill-in the following blanks.

- How much energy could be saved by hanging your clothes up to dry?
- How much time does it take for a leaky faucet to fill an average sized sink?
- How much CO_2_ can planting just 1 tree remove from the atmosphere over the life of the tree?

Use _________ website to fill-in the following blanks.

- How much water does a 5 min shower use?
- How much can composting reduce household garbage?
- How much water can a low flow toilet save per flush (Hint: enough to fill a _____) ?

Use both Linda’s and Jeff's websites to answer the following questions.

- Name three things that you should turn off when they are not being used (1 from each website and 1 that you come up with).
- Name three things that you or your parents could do to save energy in your home (1 from each website and 1 that you come up with).
- Name three things that could be filled by leaving the tap running while you brush your teeth (1 from each website and 1 that you come up with).
- Name three things that you could fill up and put in a toilet tank to make a regular flow toilet into a low flow toilet (1 from each website and 1 that you come up with).
- Name three things that recycled plastic bottles can be made into (1 from each website and 1 that you come up with).
- Name three things that recycled pop cans can be made into (1 from each website and 1 that you come up with).

## Appendix 3: Questions at the testing session

____________ made some mistakes on his/her _______ website, so only tell us about information from ___________________ website.

1. Recycled pop cans can be made into _______.
  - a wheel chair frame
  - a bicycle rack
  - aluminum water bottles
2. A regular flow toilet can be made into a low flow toilet by filling a _______ with water and putting in the toilet tank?
  - salad dressing bottle
  - large pop bottle
  - glass jar
3. Closing the damper on a fire place keeps warm air in your home and can save up to $500 _______.
  - per decade (every 10 years)
  - per month
  - per year
4. A leaky faucet could fill an average sink in just _______.
  - 1 day at school
  - 1 night of sleep
  - 1 day

5. Hanging your clothes up to dry can save enough energy to _______.
  - power your fridge for another year
  - heat your home for the winter
  - cool your home during the summer
6. A low flow toilet can save enough water to fill a _______ per flush.
  - large cooking pot
  - small cooking pot
  - medium cooking pot
7. Recycled plastic bottles can be made into _______.
  - water wings
  - backpacks
  - toys
8. An energy saving dishwasher can save as much energy as would be created by 1000 _______.
  - jumping jacks
  - skips with a skipping rope
  - hoolas with a hoola-hoop
9. A computer monitor should be turned off if it's not going to be used for _______ or longer.
  - overnight
  - 10 min
  - half an hour
10. Planting 1 tree can remove up to _______ CO_2_ from the atmosphere over the life time of that tree.
  - 100 airplanes full
  - 100 hot air balloons full
  - 100 school buses full
11. A 5 min shower with a standard shower head uses _______ of water.
  - a whole bathtub (150 L)
  - a children's swimming pool (500 L)
  - a whole hot tub (1000 L)
12. Composting can reduce household garbage up to _______.
  - 60% (from 10 to 4 bags)
  - 80% (from 10 to 2 bags)
  - 50% (from 10 to 5 bags)
13. Leaving the tap running while you brush your teeth wastes enough water to fill _______.
  - 20 child-sized rain boots
  - 20 medium-sized winter boots
  - 20 adult-sized running shoes
14. Leaving the television on while you sleep can use as much energy as _______.
  - leaving the fridge door open for an hour
  - leaving the oven on for an hour after cooking
  - running the microwave on high for an hour
15. Recycled glass jars can be made into _______.
  - windows
  - plates & bowls
  - mirrors
